# Varying Phenotypes of Leydig Cell Hyperplasia of the Ovary: Two Case Reports

**DOI:** 10.1155/2023/7178201

**Published:** 2023-08-08

**Authors:** Margaret Caulkins, Jason Ricciuti, Mohamed Desouki, Katherine LaVigne Mager

**Affiliations:** ^1^Roswell Park Comprehensive Cancer Institute, Gynecologic Oncology Department, 665 Elm Street, Buffalo, NY 14203, USA; ^2^University at Buffalo, The State University of New York, Obstetrics and Gynecology Department, Conventus, 1001 Main Street, Buffalo, NY 14203, USA; ^3^St. Luke's Hospital, Gynecologic Oncology Department, 1031 Bellevue Avenue, Suite 400, St. Louis, MO 63117, USA; ^4^Roswell Park Comprehensive Cancer Institute, Pathology Department, 665 Elm Street, Buffalo, NY 14203, USA

## Abstract

Leydig cell hyperplasia (LCH) is a rare cause of hyperandrogenism that has been described only in case reports. The cases presented herein contrast the traditional presentation of LCH with an affected asymptomatic individual. The first case involves a 74-year-old woman presenting with symptomatic hyperandrogenism, whose symptoms resolved after bilateral salpingo-oophorectomy (BSO). The second patient presented with postmenopausal bleeding and an abdominal mass. Following total abdominal hysterectomy (TAH) and BSO, pathology showed ovarian LCH with concomitant endometrial cancer. The diagnosis of LCH is complex and requires careful investigation of many differential diagnoses. Incidentally discovered LCH may shed light on evolution and disease progression. Cases of LCH found in the setting of endometrial pathology may have implications on other states of testosterone excess.

## 1. Introduction

Leydig cell hyperplasia (LCH) is a rare cause of hyperandrogenism that has been described only in case reports. Here we present two cases of LCH, with one patient experiencing symptomatic hyperandrogenism and another identified incidentally following surgery for endometrial carcinoma. These cases contrast the more traditional presentation of LCH with an asymptomatic individual. The etiology of this condition is poorly understood and disputed. The unique presentations of these cases may shed insight into the evolution of this condition.

### 1.1. Case Presentation

#### 1.1.1. Case 1

A 74-year-old woman was referred by her endocrinologist for symptomatic hyperandrogenism. Her testosterone levels had been persistently elevated for three years, with her total testosterone ranging from 134 to 177 ng/dL. The patient endorsed numerous symptoms that she attributed to the elevated testosterone, including male-pattern alopecia, worsening seborrheic dermatitis, and increased central adiposity with a protuberant, firm abdomen. She also noted mood changes, including increased anger and aggression. The patient had previously been treated with finasteride and spironolactone, with no change in symptoms.

Her past medical history was notable for type 2 diabetes mellitus, chronic kidney disease, polycystic ovarian disease, coronary artery disease, peripheral vascular disease, Sjogren's syndrome, Hashimoto's thyroiditis, basal cell carcinoma, and a non-specific disorder of mitochondrial metabolism. She reported menarche at 12 years old with regular menses throughout her life until undergoing menopause at 54 years old. The patient's daughter had a history of thyroid cancer, but otherwise no family history of cancer. Physical examination was notable for moderate alopecia. Her pelvic exam was unremarkable with no palpable masses on the bimanual examination.

The patient's primary care doctor had completed a workup before referral with no clear etiology identified. The patient had dedicated imaging, with separate magnetic resonance imaging (MRI) to evaluate the pituitary gland, ovaries, and adrenal glands, which were all normal. Her labs were also unremarkable, including normal values for 17-*α*-hydroxyprogesterone (56 ng/dL), DHEA-sulfate (21 *μ*g/dL), prolactin (2 ng/mL), sex hormone binding globulin (26 nmol/L), insulin-like growth factor-I (153 ng/mL), aldosterone (12 ng/dL), and a dexamethasone suppression test.

**Figure 1:**
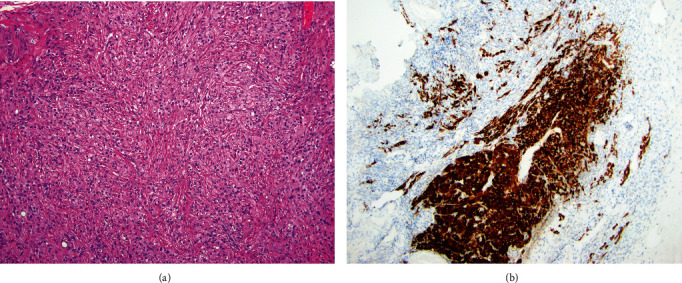
LCH. (a) The ovarian lesion is composed of large rounded/polyhedral cells with abundant eosinophilic cytoplasm, central nuclei, and prominent nucleoli. (b) The cells stain strongly positive for inhibin A by immunohistochemistry.

The patient was counseled that her symptoms were likely secondary to exogenous testosterone production in the ovaries, given her negative workup for an extra-ovarian source. She was offered a BSO given her bothersome symptoms. Hysteroscopy with dilation and curettage were also recommended, due to nonspecific fluid noted within the endometrial cavity on MRI. She underwent diagnostic laparoscopy with BSO, peritoneal biopsies, and pelvic washings as well as a hysteroscopy with dilatation and curettage. Surgery was uncomplicated, and the patient was discharged home on the same day. Intra-operative findings were notable for grossly normal ovaries and a benign-appearing cystic nodule on the peritoneum overlying the bladder, which was biopsied. Hysteroscopy demonstrated a benign appearing polyp and atrophic changes. On gross pathologic examination, the right ovary measured 2.4 cm × 1.9 cm × 1.7 cm with unremarkable, lobulated cut surfaces. The left ovary measured 2.7 cm × 2.2 cm × 1.4 cm and was grossly unremarkable. On microscopic examination, both ovaries contained large rounded/polyhedral cells with abundant eosinophilic cytoplasm, central nuclei, and prominent nucleoli predominantly in the hilar area consistent with LCH. The cells stained strongly positive for inhibin A by immunohistochemistry (Figures 1(a) and 1(b)). Patient's postoperative course was uncomplicated. Two months after surgery, her total testosterone had normalized to 13 ng/dL and she reported improved symptoms, including decreased hair loss, less aggression, and improved acne.

#### 1.1.2. Case 2

A 55-year-old female was referred for postmenopausal bleeding and an abdominal mass. She was diagnosed with a 16 cm pelvic mass, likely degenerating fibroid, on ultrasound and computerized tomography (CT) after her gynecologist noted a palpable mass on a routine examination. The patient endorsed new constipation, early satiety, and bloating for several months before presenting for care. She also reported vaginal spotting over the last year that had become increasingly heavy over recent months.

Her past medical history was notable for class III obesity (BMI 50.7), obstructive sleep apnea, chronic pain syndrome, and polysubstance abuse. She reported menarche at 14 years old and menopause at 50 years old.

On physical examination, there was scant blood in the vagina and no palpable masses on the bimanual examination (limited by body habitus). An endometrial biopsy was obtained and demonstrated FIGO grade 1 endometrioid adenocarcinoma. The patient had a repeat CT of the chest, abdomen, and pelvis, which showed an increase in the size of the pelvic mass to 17.8 cm.

**Figure 2:**
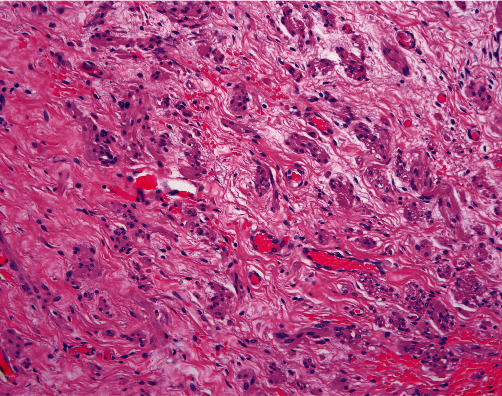
LCH. The left ovary contains large rounded/polyhedral cells with abundant eosinophilic cytoplasm, central nuclei, and prominent nucleoli in the hilar area.

She underwent a total abdominal hysterectomy, BSO, and prolonged lysis of adhesions. Final pathology reported FIGO grade 2 endometrioid carcinoma with a microcystic, elongated, and fragmented pattern of invasion and involvement of the cervical stroma. The left ovary grossly measured 3.5 cm × 2.4 cm × 1.7 cm with a subserosal cyst that had smooth outer and inner linings. On microscopic examination, the left ovary contained large rounded/polyhedral cells with abundant eosinophilic cytoplasm, central nuclei, and prominent nucleoli in the hilar area, consistent with LCH (Figure 2). An incidental serous cystadenoma was also identified. Following a complicated postoperative recovery due to her multiple medical comorbidities, she underwent adjuvant radiation therapy for her endometrial cancer and was then started on surveillance.

## 2. Discussion

LCH has previously been described in case reports as a rare cause of postmenopausal hyperandrogenism. Our first case provides a classic example of the typical LCH presentation with symptoms and laboratory findings consistent with excess androgen production. Some level of hyperandrogenism can be considered a normal physiologic response to a menopause-induced drop in estrogen with a lagging concomitant decline in androgen production [1]. Symptomatic patients should undergo an evaluation of the adrenal glands, pituitary gland, and ovaries. The differential is broad for these patients, including androgen-producing tumors of the ovary or adrenals, Cushing's syndrome, obesity, congenital adrenal hyperplasia, and iatrogenic causes [1].

One of the most common causes of hyperandrogenism in females is the familiar polycystic ovarian syndrome (PCOS). The first patient in this series had a history of PCOS, with worsening hyperandrogenic symptoms after menopause. While PCOS can be associated with mild testosterone elevations in postmenopausal women, the virilization symptoms reported by the patient were atypical. As demonstrated by this patient, evolving or severe symptoms must be scrutinized further. Though they comprise less than 5% of all ovarian tumors, androgen-secreting neoplasms of the ovary should be considered in patients with virilization [2, 3]. Included in this group are Leydig hilus cell tumors, nonhilar Leydig cell tumors, Sertoli-Leydig cell tumors, stromal luteomas, steroid cell tumors not otherwise specified, and ovarian hyperthecosis [2, 4].

Evaluation of symptomatic patients often starts with quantification of androgen levels and testing for hypercortisolism. This is traditionally followed by imaging of the adrenal glands, pituitary gland, and ovaries, with additional tests as warranted based on results [1]. The extent of testing for postmenopausal women with symptoms of hyperandrogenism has been contested by some authors. For example, Taylor et al. argued that BSO should be performed in patients who desire treatment, without extensive endocrine testing or search for metastatic disease, as long as there is no pronounced adrenal tumor or ovarian enlargement on imaging [5].

The cases presented herein provide an excellent contrast of the varied presentations and diagnostic challenges presented by this condition. Our second case had abnormal imaging, however, it was ultimately unrelated to her incidental diagnosis of LCH. Though this patient's LCH was not causing disturbing symptomatology, these distinct presentations of LCH could provide insight into different etiologies of this rare condition. While the pathophysiology of LCH is ultimately unknown, several hypotheses have been presented. Leydig cells typically originate in the ovarian hilus, near the nonmedullated nerves, and are seen in almost all postmenopausal women [5]. Hilar cell production of androgens in response to the presence of human chorionic gonadotropin (hCG) has been confirmed in vitro [6, 7]. It has been hypothesized that hCG activity, which is increased during menopause, may trigger the proliferation of these hilus cells and lead to hyperandrogenism [7, 8, 9]. This supposition is supported by the finding of LCH in postmenopausal patients with hyperandrogenism, however, it does not account for all cases, such as our second patient. [8, 9, 10, 11] It has also been postulated that hyperplasia may be triggered in response to increased pressure from adjacent ovarian tumors or cysts [12, 6, 13]. There have been at least five case reports of LCH in association with serous cystadenomas or fibromas. This may suggest a possible etiology for our second case, in which LCH was found in association with a serous cystadenoma. The association between LCH and ovarian masses in asymptomatic patients may suggest a unique etiology and a separate disease process from the traditional hypersecretory LCH. Alternatively, these cases could lend an insight into the natural progression of LCH, with a long interval between initial stretch injury leading to LCH and the hyperandrogenic secretory phase typically seen at presentation.

The second case presented is additionally unique for its association with uterine pathology. While some hormonally active ovarian tumors are known to be associated with endometrial hyperplasia or carcinoma, to the best of our knowledge, our second case is the first description of endometrial carcinoma found in conjunction with LCH [14, 15]. While this uterine pathology may have been correlative, there is a possibility of causality. The excessive levels of testosterone produced by LCH may have spurred excessive aromatization, leading to a state of unopposed estrogen. The possibility of a causative relationship has implications for other states of testosterone excess, such as transmasculine patients on testosterone supplementation, who are transitioning from female to male (FTM). Mechanistically, this risk is credible, however, a causal relationship has limited supporting data. Currently, there are no uterine cancer screening guidelines for these patients, due to the paucity of data supporting the need for screening. There has been only one case report of endometrial cancer in the context of an FTM trans patient [16]. There have additionally been two small studies that imply that the excess testosterone states seen in these patients induce endometrial atrophy, implying a low estrogen state [17, 18]. The varying endometrial responses between LCH patients and trans patients on exogenous testosterone may imply a difference in endometrial response based on longevity of exposure or even volume of exposure. Regardless, this association may at least support endometrial sampling in patients with LCH, especially in light of the low risk associated with endometrial sampling.

## 3. Conclusion

LCH represents a rare cause of hyperandrogenism in females. The diagnosis of this rare condition is complex and requires careful investigation of a long list of differential diagnoses. Furthermore, research is needed to determine the most effective diagnostic algorithm. Treatment is successful with BSO, with resolution of symptoms. However, this rare condition can also be found incidentally in the asymptomatic female or association with other gynecologic pathology. Incidentally discovered LCH may shed light on evolution and disease progression. Cases of LCH found in the setting of endometrial pathology may have implications on other states of testosterone excess.

## Data Availability

Data supporting this research article are available from the corresponding author or first author on reasonable request.

## References

[1] Koeneman M. M., Heiligers-Duckers C., van der Velde R. (2016). Ovarian Leydig cell hyperplasia as a rare cause of hair loss in a postmenopausal female patient: a case report and diagnostic approach toward postmenopausal Hyperandrogenism. *European Journal of Obstetrics and Gynecology and Reproductive Biology*.

[2] Gandrapu B., Sundar P., Phillips B. (2018). Hyperandrogenism in a postmenopausal woman secondary to testosterone secreting ovarian stromal tumor with acoustic schwannoma. *Case Reports in Endocrinology*.

[3] Delibasi T., Erdogan M., Serinsoz E., Kaygusuz G., Erdogan G., Sertcelik A. (2007). Ovarian hilus-cell hyperplasia and high serum testosterone in a patient with postmenopausal virilization. *Endocrine Practice*.

[4] Udhreja P. R., Banerji A., Desai D. P., Vaishnani J. B. (2014). Androgen—secreting steroid cell tumor of the ovary. *Indian Journal of Pathology and Microbiology*.

[5] Taylor H. C., Pillay I., Setrakian S. (2000). Diffuse stromal Leydig cell hyperplasia: a unique cause of postmenopausal hyperandrogenism and virilization. *Mayo Clinic Proceedings*.

[6] Hayes F. J., Sheahan K., Rajendiran S., McKenna T. J. (1997). Virilization in a postmenopausal woman as a result of hilus cell hyperplasia associated with a simple ovarian cyst. *American Journal of Obstetrics and Gynecology*.

[7] McLellan A. R., Mowat A., Cordiner J., Beastall G. H., Wallace A. M. (1990). Hilus cell pathology and hirsutism. *Clinical Endocrinology*.

[8] Kim Y., Lee I. O., Chung J. E. (2018). Incidental finding of a Sertoli-Leydig cell tumor in a postmenopausal woman with complex endometrial hyperplasia. *International Journal of Reproduction, Contraception, Obstetrics and Gynecology*.

[9] Mehta J. M., Miller J. L., Cannon A. J., Mardekian S. K., Kenyon L. C., Jabbour S. A. (2014). Ovarian Leydig cell hyperplasia: an unusual case of virilization in a postmenopausal woman. *Case Reports in Endocrinology*.

[10] Sternberg W. H., Roth L. M. (1973). Ovarian stromal tumors containing Leydig cells. I. Stromal-Leydig cell tumor and non-neoplastic transformation of ovarian stroma to Leydig cells. *Cancer*.

[11] Tutzer M., Winnykamien I., Guardia J. D., Castelo-Branco C. (2014). Hyperandrogenism in post-menopausal women: a diagnosis challenge. *Gynecological Endocrinology*.

[12] Braithwaite S. S., Bitterman P., DeGeest K., Lebbin D. (2001). Postmenopausal virilization, simple ovarian cyst, and hilus cell hyperplasia—is there an association?. *Endocrine Practice*.

[13] Yoon B. S., Seong S. J., Park C. T., Park H., Shim J. Y., Kim J. Y. (2010). Cellular fibroma of the ovary containing Leydig cell hyperplasia: a case report. *Journal of Gynecologic Oncology*.

[14] Dunnihoo D. R., Grieme D. L., Woolf R. B. (1966). Hilar-cell tumours of the ovary. *Obstetrics and Gynaecology*.

[15] Hofland M., Cosyns S., Sutter P. D., Bourgain C., Velkeniers B. (2013). Leydig cell hyperplasia and Leydig cell tumour in postmenopausal women: report of two cases. *Gynecological Endocrinology*.

[16] Urban R. R., Teng N. N. H., Kapp D. S. (2011). Gynecologic malignancies in female-to-male transgender patients: the need of original gender surveillance. *American Journal of Obstetrics and Gynecology*.

[17] Grynberg M., Fanchin R., Dubost G. (2010). Histology of genital tract and breast tissue after long-term testosterone administration in a female-to-male transsexual population. *Reproductive Biomedicine Online*.

[18] Perrone A. M., Cerpolini S., Salfi N. C. M. (2009). Effect of long‐term testosterone administration on the endometrium of female‐to‐male (FTM) transsexuals. *The Journal of Sexual Medicine*.

